# Factors influencing the implementation of fall-prevention programmes: a systematic review and synthesis of qualitative studies

**DOI:** 10.1186/1748-5908-7-91

**Published:** 2012-09-14

**Authors:** Sue Child, Victoria Goodwin, Ruth Garside, Tracey Jones-Hughes, Kate Boddy, Ken Stein

**Affiliations:** 1 PenCLAHRC, University of Exeter Medical School, University of Exeter, Exeter, UK; 2 European Centre for Environment and Human Health, University of Exeter Medical School, University of Exeter, Exeter, UK; 3 Knowledge Spa, Royal Cornwall Hospital, Truro, UK; 4 PenCLAHRC, University of Exeter Medical School, University of Exeter, Exeter, UK

**Keywords:** Fall prevention, Systematic review, Meta-ethnography, Implementation

## Abstract

**Background:**

More than a third of people over the age of 65 years fall each year. Falling can lead to a reduction in quality of life, mortality, and a risk of prolonged hospitalisation. Reducing and preventing falls has become an international health priority. To help understand why research evidence has often not been translated into changes in clinical practice, we undertook a systematic review and synthesis of qualitative research in order to identify what factors serve as barriers and facilitators to the successful implementation of fall-prevention programmes.

**Methods:**

We conducted a review of literature published between 1980 and January 2012 for qualitative research studies that examined barriers and facilitators to the effective implementation of fall-prevention interventions among community-dwelling older people and healthcare professionals. Two reviewers independently screened studies for inclusion, extracted data, and assessed methodological quality according to predefined criteria. Findings were synthesised using meta-ethnography.

**Results:**

Of the 5010 articles identified through database searching, 19 were included in the review. Analysis of the 19 studies revealed limited information about the mechanisms by which barriers to implementation of fall-prevention interventions had been overcome. Data synthesis produced three overarching concepts: (1) practical considerations, (2) adapting for community, and (3) psychosocial. A line of argument synthesis describes the barriers and facilitators to the successful implementation of fall-prevention programmes. These concepts show that the implementation of fall-prevention programmes is complex and multifactorial. This is the first systematic review and synthesis of qualitative studies to examine factors influencing the implementation of fall-prevention programmes from the perspectives of both the healthcare professional and the community-dwelling older person.

**Conclusions:**

The current literature on barriers and facilitators to the implementation of fall-prevention programmes examines a variety of interventions. However, the ways in which the interventions are reported suggests there are substantial methodological challenges that often inhibit implementation into practice. We recommend that successful implementation requires individuals, professionals, and organisations to modify established behaviours, thoughts, and practice. The issues identified through this synthesis need to be fully considered and addressed if fall-prevention programmes are to be successfully implemented into clinical practice.

## Background

More than a third of people over the age of 65 years fall each year [1], resulting in a spiral of negative outcomes, including functional decline, reduced quality of life, morbidity, mortality, and risk of prolonged hospitalisation [1,2]. As a result, reducing falls has become an international health priority [3].

During the past 20 years, growing awareness of the serious consequences of falls has led to the development and evaluation of many fall-prevention programmes. Programmes have been described in a taxonomy of interventions [4] and may include an assessment, in addition to interventions such as exercise, medication, environmental modification, and knowledge.

However, the potential impact of such programmes is often constrained by barriers to their effective implementation [5]. Despite the publication of randomised controlled trials [6-8] and clinical guidelines [9,10] showing that fall-prevention interventions can be successful, evidence from research has often not been translated into changes in clinical practice [11]. As a result, falls and fall-related injuries continue to rise, along with associated healthcare costs. For example, in the United Kingdom, fragility fractures account for approximately £2 billion a year [12].

It is recognised that both older people and clinicians may be required to modify established ways of living and working in order to facilitate and embrace new attitudes and behaviours that may result in fewer falls. For example, healthcare professionals might be encouraged to incorporate evidence-based falls risk assessment into routine clinical practice [13,14].

We have previously reported a systematic review of studies examining the quantitative evaluation of implementation strategies to reduce falls [15]; however, these study designs are unable to explore factors that influence the effects of implementation strategies. Therefore, the aim of this review is to identify key factors that act as barriers and facilitators to the effective implementation of evidence-based best practice in relation to the prevention of falls among community-dwelling older people.

## Methods

### Design

We conducted a systematic literature search; further, quality appraisal of the included studies and synthesis using a meta-ethnographic approach based on the methods by Noblit and Hare [16] was undertaken. The synthesis was also informed by recent meta-ethnographies that assessed older people’s views in relation to the risk of falling [17,18]. The aim of such a synthesis is to identify unifying features common to, or disputed across, a number of sources and to create new findings.

### Search strategy and selection criteria

An information scientist (KB) and reviewers (VG, TJH) devised a search strategy of key studies examining factors affecting the implementation of fall-prevention programmes (Additional File 1). The study protocol has been published by the Peninsula Collaboration for Applied Health Research and Care website ( http://clahrc-peninsula.nihr.ac.uk/implementation-of-falls-prevention-programmes.php). The search strategy was adapted and run in the following electronic databases for the period January 1980 to January 2012: AMED and CINAHL (using the EBSCO interface), Cochrane Database of Systematic Reviews, CENTRAL, Medline, Embase and PsychInfo (using the OVID interface), and the Social Sciences Citation Index. Two reviewers (VG and TJH) independently screened all titles and abstracts. Studies were included if they examined influences on the implementation of fall-prevention programmes among community-dwelling older adults and used recognised qualitative methods of data collection and analysis. Editorials, opinion papers, and studies only reported as conference abstracts were excluded. Only papers published in the English language were included in the review. Studies that did not fulfil the criteria were excluded and their bibliographic details listed with reason for exclusion. Any discrepancies were resolved by consensus and, where necessary, a third reviewer was consulted (KS or SC).

### Study quality assessment

Data abstraction was performed by two independent reviewers (VG, SC). The following information was extracted: study location and setting, population, recruitment strategy, sample size, method of investigation, nature of the fall-prevention intervention, method(s) of analysis, and study findings. We used a structured approach to describe the quality of the included studies using criteria developed by Wallace *et al.*[19].

While reading and rereading the papers, we used thematic analysis [20] to identify recurrent issues arising in the studies. We extracted quotes relating to barriers and facilitators to fall-prevention interventions, which were later tabulated in order to develop first- and second-order concepts from each paper. We then developed a coding scheme to code the original author interpretations and build tabular comparisons in order to develop third-order concepts. Meta-ethnography was used to synthesise the data from the included studies [16].

## Results

**Figure 1:**
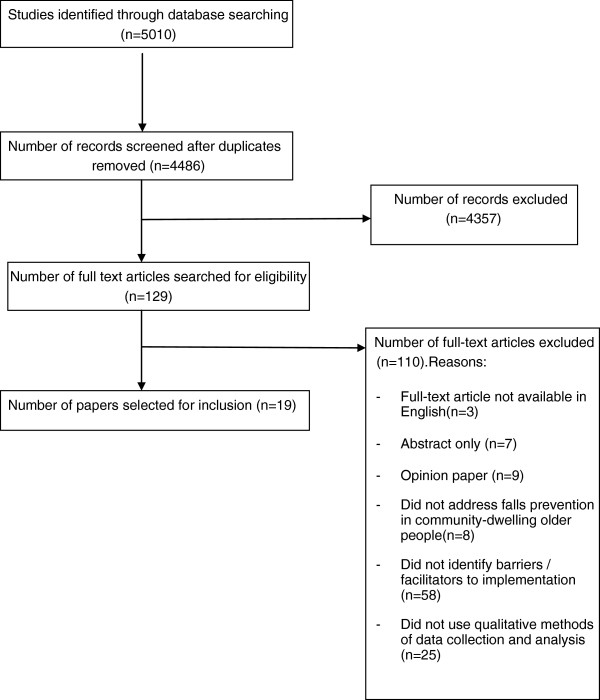
Flowchart summarizing the screening process.

The initial search criteria yielded 4486 potential papers after the removal of duplicates (Figure 1). Following completion of screening, a total of 19 studies were included in the systematic review.

Study characteristics and the methodological approach of each of the studies is presented in Table 1, with quality assessments described in Additional File 2 Six studies examined barriers and facilitators to implementation of fall-prevention programmes from the perspective of healthcare professionals [13,14,21-24], 12 from the experiences of community-dwelling older adults [25-36], and 1 examined perspectives from both patients and healthcare workers in a newly established falls clinic [37]. The studies were international, with two based in Canada [24,25], four in the United States [13,14,22,33], six in the UK [27,29,31,34-36], one in Australia [23], two in Denmark [28,37], one in Norway [32], and one in New Zealand [30]. Two studies involved participants from a number of different countries [21,26].

**Table 1:** Details of included qualitative studies of barriers and facilitators to fall-prevention interventions

**Study location**	**Study aim and type of fall-prevention intervention addressed**	**Stated methodology**	**Method of investigation. Analysis method.**	**Setting, sample size, and strategy (where specified in the study) and characteristics**
Aminzadeh and Edwards 1998 (Canada) [25]	To explore views of older people on the use of assistive devices.	Focus groups	Four focus group interviews. Thematic analysis based on methods by Krueger.	Convenience sample of community-dwelling older people.
				n = 30 (Italian and British-Canadian)
				n = 21 women; n = 9 men. Mean age 72.2 (range = 61–86); 16 lived alone.
Baker *et al.*, 2005 (USA) [13]	To report on barriers and facilitators to incorporate evidence-based fall risk assessment into clinical practice in a defined geographical area. Multiple professional behaviour-change interventions were used to encourage providers to incorporate evidence-based fall assessment into practice.	Discourse analysis	Semistructured interviews.	Healthcare providers who agreed to receive outreach visits as part of the Connecticut Collaboration for Fall Prevention (CCFP) programme. n = 119 rehabilitation facilities; n = 125 primary care offices; n = 7 hospitals; n = 26 home care agencies.
			Discourse analysis of interviews.	
Bell and Stirling 2006 (Australia) [23]	To explore the development of ‘whole-of-patient’ approaches fall intervention by the implementation of a quick screen fall risk assessment tool in general practice by registered nurses.	Semistructured interviews	10 semistructured interviews.	Registered practice nurses implementing a Clinical Falls Assessment Tool in 10 general practices (n = 10) in Tasmania.
			Textual hermeneutic analysis (interpretation of principal themes).	
Chou *et al.*, 2005 (USA) [22]	To identify barriers and facilitators to the implementation of fall risk management in primary care through academic (education) outreach visits with primary care providers.	Semistructured interviews	18 semistructured interviews.	Primary care physicians who had attended an educational outreach visit as part of CCFP programmes within last three months; n = 18.
			Constant comparative method based on Glaser and Strauss.	
De Groot and Fagerstrom 2011 (Norway) [32]	To describe the motivating factors and barriers for older adults to adhere to group exercises in the local community that aim to prevent falls and thereby gain further knowledge about how health professionals can stimulate adherence.	Semistructured interviews	10 semistructured interviews.	10 adults; 5 men and 5 women who had previously been part of an exercise intervention project. Mean age 83. Equal representation of those afraid of falling and those who were not.
			Descriptive content analysis.	
Dickinson *et al.*, 2011 (UK) [34]	To explore older people’s perceptions of the facilitators and barriers to participation in fall-prevention exercise interventions in the United Kingdom. The study included participants who had taken part or declined to take part in postural stability, Tai Chi, and general exercise classes.	Focus groups and semistructured interviews	17 focus groups and 65 semistructured interviews.	187 older people who had previously attended fall-prevention interventions in four geographical areas in the South of England. Caucasian (n = 125; men = 35, women = 90; mean age 77.6). Asian (n = 32; men = 6, women = 26; mean age 69.7). Chinese (n = 30; men = 9, women = 21; mean age 75).
				17 focus groups (n = 122 attending). Interviews (n = 65).
			Constant comparative method drawn from grounded theory.	
Evron *et al.*, 2009a (Denmark) [37]	To describe the social processes affecting the implementation of new strategies in fall management through health education.	Interviews, participant observation, and document analysis	28 semistructured interviews and participant observation.	Convenience sample of staff working in a falls assessment clinic. Interviews with key informants in hospitals, n = 6; key informants from rehabilitation clinic, n = 4; key informants from municipality, n = 2; *ad hoc* informants (healthcare professionals, patients encountered during participant observation), n = 16.
			Thematic analysis.	
Evron *et al.*, 2009b (Denmark) [28]	To gain new knowledge about barriers to participation in hospital-based fall assessment and interventions, including exercise.	Semistructured interviews	20 semistructured interviews.	Convenience sample of community-dwelling older people attending a falls assessment clinic. n = 10 refusers: n = 8 women, n = 2 men, mean age 81 years (range = 70–87); n = 10 acceptors: n = 8 women, n = 2 men, mean age 86 years (range = 78–94).
			Thematic analysis.	
Fortinsky *et al.*, 2004 (USA) [14]	To establish how far an educational intervention helps healthcare providers address evidence-based fall risk factors and determine barriers to implementation.	Structured interviews	33 structured interviews.	Healthcare professionals participating in the CCFP programme. n = 22 women; n = 11 men; mean age 46 (range = 28–63).
			Thematic analysis.	
				n = 5 emergency dept physicians; n = 10 hospital discharge planners; n = 10 home health agency nurses; n = 8 office-based primary care physicians.
Hanson and Salmoni, 2011 (Canada) [24]	To identify stakeholders’ perceptions of sustainability after the completion of a community-based fall-prevention education project in three communities in Ontario.	Holistic, multiple case study method	45 semistructured interviews following a focussed interview format and using open-ended questions.	Key stakeholders involved in components of fall-prevention programmes in three Ontario communities. n = 18 community one; n = 15 community two; n = 12 community three.
			Interview analysis using pattern matching and explanation building aided by NVivo software.	
Hawley 2009 (UK) [31]	To explore what might encourage older people to exercise at home after falls rehabilitation.	Grounded theory	Nine unstructured interviews.	Community-dwelling older people who had participated in a fall-prevention programme; >60 years of age.
			Grounded theory.	
Horne *et al.*, 2009 (UK) [29]	To identify salient beliefs that influence uptake and adherence to exercise for fall prevention among different ethnic communities.	Ethnographic	15 focus groups, 40 semistructured interviews.	Purposive sample of community-dwelling older people with different experiences of participation or nonparticipation in exercise. Recruited through fieldwork.
			Framework analysis.	
				Focus groups n = 87 (n = 58 Caucasian; n = 44 women; n = 14 men; mean age 65.4).(n = 29 South Asian; n = 13 women; n = 16 men; mean age 66.1 years).
				Interviews n = 40 (n = 14 Caucasian women; n = 9 Caucasian men; mean age 64.8; n = 10 South Asian women; n = 7 South Asian men; mean age 65.2).
Horton and Dickinson, 2011 (UK) [35]	To explore the perceptions about the use of physical activity in older Chinese people, living in England, and identify barriers and facilitators to exercise uptake.	Grounded theory	Two focus groups, 10 in-depth interviews.	Purposive sample of 30 Chinese community-dwelling older people who attended Tai Chi classes (male = 9, female = 21, mean age 70.2). Focus groups n = 20 (10 in each group). Interviews n = 10.
			Constant comparative analysis.	
Hutton *et al.*, 2009 (New Zealand) [30]	To identify factors that older adults feel help or hinder their involvement in exercise classes.	Focus groups	Five focus groups.	Community-dwelling older people identified at risk of falling who had participated in a randomised controlled trial of Tai Chi intervention classes. Focus groups = 20 participants aged 68–81 years.
			Thematic analysis.	
Mackenzie 2009 (Australia, UK, Canada) [21]	To identify how educating health professionals about home hazard reduction improves the implementation of home modification fall-prevention programmes in the community.	Focus groups and semistructured interviews	10 focus groups (n = 2 Australia, n = 4 in Canada, n = 4 in UK).	Healthcare professionals using HOME FAST falls and accident screening tool. Occupational therapists n = 30; occupational therapy assistants n = 2; nurses n = 10; physiotherapists n = 3; paramedics n = 2; geriatricians n = 1; social worker n = 1; consumer organisation representative n = 1.
			50 semistructured interviews.	
			Constant comparative analysis.	
Nahm *et al.*, 2009 (USA) [33]	To ascertain the impact of the social cognitive theory-based structured hip fracture prevention website (TSW) on health behaviours through peer education.	Online, randomised controlled study—part of an exploratory, qualitative study	Content analysis of discussion board postings.	Convenience sample of 116 participants from 245 people (77.6%) who had posted thoughts about falling on online discussion boards. All participants were >55 years, community-dwelling, English speaking who had access to, and working knowledge of, the internet and email access either at home or in the community.
Stewart and McVittie, 2011 (UK) [36]	To examine the psychological experiences of involvement in a multidisciplinary educational falls-prevention programme.	Semistructured interviews	Eight semistructured interviews.	Purposive sample of eight housebound, community-dwelling older people who had participated in a multidisciplinary fall-prevention programme (n = 1 male, n = 7 female). Mean age 84 years. All participants were of Scottish (European) background.
			Interpretative phenomenological approach (IPA).	
Vernon and Ross, 2008 (UK) [27]	To explore the barriers to access and acceptability to participation in community-based exercise classes for fall prevention.	Mixed qualitative	22 open interviews.	Community-dwelling older people who had attended a community balance class. n = 20 women; n = 2 men; age range 65–94. Black British Caribbean n = 4; White Irish n = 2; White British n = 16.
			Questionnaires (only interview analysis reported in the paper).	
			Three focus groups.	
Yardley *et al.*, 2006 (Denmark, Germany, Greece, Switzerland, The Netherlands, UK) [26]	To identify barriers and facilitators to the uptake of various fall-prevention interventions, including exercise and home modifications.	69 semistructured interviews	Interviews.	Community-dwelling older people who had declined or participated in fall-prevention interventions. n = 19 men; n = 50 women. Age ranges 68–97 across six European countries (50% of the participants had previously fallen).
			Framework and content analysis.	

A multitude of different methods of analysis had been chosen by the authors to examine data. Five studies [14,25,28,30,37] used thematic analysis, two studies used framework analysis [26,29], and one used grounded theory [31]. Four studies used constant comparative analysis [21,22,34,35], and one used a method described by the authors as hermeneutic textual analysis [23]. One study used discourse analysis [13], two employed descriptive content analysis [32,33], two used interview analysis [24,27], and one used interpretative phenomenological analysis [36].

Three overarching reviewer concepts were identified by reading and rereading the selected papers to enable synthesis of findings across the studies. These were (1) practical considerations, (2) adapting for community, and (3) psychosocial. Two concepts (practical considerations and psychosocial) have a number of subthemes within the overall concept. In order to help the structure of the systematic review, we have chosen to discuss each overarching category and subtheme separately. However, we acknowledge that this format may suggest each category exists in isolation, whereas in reality they all have interchangeable boundaries. Table 2 shows the contribution of included studies to key concepts and synthesis.

**Table 2:** Contribution of included studies to key concepts and synthesis

**Study**	**Practical considerations**			**Adapting for community Social and cultural**	**Psychosocial**	
	**Economic**	**Access to intervention**	**Time**		**Transforming identities**	**Defining the expert**
**Aminzadeh & Edwards, 1998**[25]	**X**		**X**	**X**	**X**	**X**
**Baker *et al.*, 2005**[13]	**X**		**X**			**X**
**Bell & Stirling, 2006**[23]	**X**		**X**		**X**	**X**
**Chou *et al.*, 2005**[22]	**X**	**X**	**X**	**X**		**X**
**De Groot & Fagerstrom, 2010**[32]	**X**	**X**	**X**	**X**		**X**
**Dickinson *et al.*, 2011**[34]				**X**		**X**
**Evron *et al.*, 2009a**[37]	**X**					**X**
**Evron *et al.*, 2009b**[28]	**X**	**X**	**X**			**X**
**Fortinsky *et al.*, 2004**[14]	**X**	**X**	**X**	**X**	**X**	
**Hanson & Salmoni, 2011**[24]	**X**		**X**			
**Hawley 2009**[31]	**X**			**X**	**X**	**X**
**Horne *et al.*, 2009**[29]				**X**	**X**	
**Horton & Dickinson, 2011**[35]				**X**		
**Hutton *et al.*, 2009**[30]		**X**	**X**			
**Mackenzie 2009**[21]	**X**		**X**		**X**	**X**
**Nahm *et al.*, 2009**[33]				**X**		**X**
**Stewart & McVittie, 2011**[36]			**X**	**X**	**X**	
**Vernon & Ross, 2008**[27]	**X**	**X**		**X**		**X**
**Yardley *et al.*, 2006**[26]	**X**	**X**	**X**	**X**	**X**	**X**

### Practical considerations

In this section, we examine three practical considerations that need to be addressed in designing and implementing fall-prevention interventions: economic factors (cost), access to the intervention, and time.

It may be argued that these considerations are generic across all implementation programmes *per se*, and a further discussion here would offer very little in the way of generating new knowledge. Yet the fact that issues surrounding cost, time, and the availability of fall-prevention interventions have arisen from our meta-ethnography might suggest that earlier findings surrounding these generic considerations have yet to be dealt with satisfactorily.

### Economic factors

 Thirteeen studies discuss the economic costs involved in the implementation of fall-prevention interventions [13,14,21-28,31,32,37].

For the individual, there may be financial costs associated with the purchase of assistive devices [25] and transportation to and from fall-prevention interventions (such as exercise classes), alongside fees for attendance [22,26,28,31]. It would appear to be an overriding assumption that all community-dwelling older people have the financial means to participate fully in fall-prevention interventions [31], yet this may not be the case, and the types of financial costs considered above may be prohibitive and serve as barriers to attendance. However, there appeared to be a general consensus amongst older people that the cost of an intervention was not perceived to be a barrier to participation, as long as the cost was ‘reasonable’ enough [35]:

She [the physiotherapist] gave me an exercise programme…I would like to join a training centre…but with my income I have to look wistfully at that.—Respondent [28].

In countries where medication is not provided free of charge, the out-of-pocket cost of drugs might be prohibitive for the older person. Despite the fact that by not controlling poor balance or stopping constant urinary infections, the older person may be identified to have an increased risk of falling, stark choices sometimes had to be made about which medications are able to be afforded:

These people have to pay for their drugs, so sometimes they will actually come in and say, ‘Do I really need to take such and such?’—Physician [22].

At an organisational level, healthcare providers such as fall-prevention clinics have found that the diversity of both national funding and private medical insurance schemes have impacted their ability to undertake comprehensive individual fall risk assessments and refer onwards to an appropriate intervention [13]. This appears to be of greater significance in the United States, where older people are able to choose from a number of different insurance schemes that offer considerable variance in coverage and payment for different aspects of fall prevention [13]. Three of the included studies based in the United States [13,14,22] indicate that many healthcare professionals perceived that the time required to undertake a full fall risk assessment was inadequately reimbursed through private healthcare providers, and the pressures to meet financial and time obligations was commonly cited as being a barrier to offering a full fall risk assessment:

Fall prevention might take a long time and you don’t get reimbursed at all for any of the fall-prevention counselling you do.—Healthcare professional [22].

However, issues of cost are not confined solely to the United States. Healthcare professionals across the studies more widely also cited that lack of adequate reimbursement for falls assessment and associated paperwork were key barriers to recommending and implementing appropriate fall-prevention interventions [14,22,23,37]. In both Australia and the United Kingdom, fall-prevention funding often focussed on the secondary prevention of falls rather than preventing falls occurring in the first place [21]. There appears to be an urgent need to increase available resources in order to provide much-needed extra staff, as well as to improve training for all healthcare professionals in fall-prevention interventions and risk assessment [21]. A similar pattern was evident in Denmark, where competition between different professionals and departments for the same resources had diluted the amount of money available across all providers for fall-prevention services [37].

For societies more generally, dealing with falls is increasing the burden of healthcare costs [14]. Despite this, there is often a lack of a national mandate within countries to coordinate fall-prevention interventions [13]. In turn, there are limitations within healthcare systems, with inadequate access to suitably trained staff for fall-prevention services [14]. Expertise appears to be compromised as professionals now compete for the same resource [37]. Whatever fall-prevention interventions are offered, they need to be sustainable and there needs to be strategic planning to ensure that monies are available for the lifetime of the programme components:

I’m hoping that they are going to be ongoing but when there is not the direct money, when there is not the direct resources, many, many, many other priorities evolve and take precedence.—Stakeholder in community-based fall-prevention programme [24].

### Access to intervention

The ease of access to a fall-prevention intervention appears to facilitate successful implementation [14,22,26-28,30,32]. Driving independence clearly facilitated participation:

Yes because many can’t…you don’t get anywhere because you don’t drive a car, and you don’t have the possibility to…they can’t walk if it’s too far..—Participant [32].

Access to the intervention is not only affected by ability to drive, availability, and cost of transport but also by travelling distance, car parking facilities, and perceived seasonal constraints on driving. Seasonal influence would appear to be of greater significance in countries that experience harsher and more prolonged winters. A long period of snow and ice could heighten fear of falling among community-dwelling older people, thereby serving to restrict outdoor movement and travel:

…this winter there’s been so much snow. Then I don’t go out. Because I’m afraid of the snow and the ice..—Respondent from Norway [32].

Public transport also poses several barriers to participation. For example, one study based in New Zealand highlighted that when bus schedules did not directly align with class times, attendance at fall-prevention interventions was substantially reduced [30]. A UK study [27] highlighted that when the location of an exercise class was changed, which then necessitated a longer journey by public transport, many older people were deterred from participating. The cost of public transport or taking a private taxi is also a prohibiting factor for some older people:

I don’t think I could go by taxi because when I read the newspaper I get sick. How many millions did the public spend on taxis?—Respondent [32].

### Time

Time also impacts the success or failure of fall-prevention interventions from a number of different perspectives [13,14,21-26,28,30,32,36]. For example, the daily routines of older people appear to be often disregarded in favour of the healthcare professional [30], whose productive use of time appears to have greater priority:

I know they will pick me up and bring me back home, but I think it is a matter of time. Being ready and waiting – all together it takes a long time and I don’t have the energy for that..—Refuser in fall-prevention classes [28].

Once I waited two hours for the driver. I don’t take that so serious… it is something you have to put up with [in the healthcare system].—Acceptor of fall-prevention intervention [28].

A perceived lack of time remains a significant factor for all staff working within healthcare organisations. Concerns about the best use of time often appeared central to the decision about whether or not to undertake a comprehensive fall assessment [22,23]:

I don’t have the time to do that right now. I really don’t get the time to sit down with each patient and say ‘Right, I’m going to case manage this person and make sure that every aspect of their health and well-being is taken care of’. Because as long as we see these people, we see them for 15 min, 20 min, and then they’re gone.—Practice Nurse [23].

The shortage of time meant difficulties with adding fall-prevention services into already overloaded patient encounters, especially with patients with already impaired mobility [14,21,22]:

There is so much paperwork to be done in order for a nurse or social worker to go out and assess fall safety. Somebody has to hold them [patients attending fall assessment], and it just ties down a person for 15 min, which in the middle of the day we don’t have.—Physician [22].

Time pressures also impacted on more personal care within the home environment. Even when a specific need had been identified, such as someone needing help with meals, face-to-face encounters with social care workers were often rushed:

You’re just a number. Say, for instance, if you were able to make your coffee, you’d maybe have your sandwich and have your coffee later; well, everything’s put in front of you. It’s like being in a home, ‘There’s your meal, take it.’ Eat it or lump it.—75-year-old woman with severe osteoporosis [36].

### Adapting for community

This section examines the different social and cultural influences on the use (and acceptability) of assistive devices, types of exercise, and fatalistic attitudes towards falling evident in different communities. Specific cultures examined were Italian and British Canadians [25], Caucasian [29,34], Asian [29,34], Chinese [34,35], Scottish [36], Black British Caribbean, White Irish, and White British [27]. A recurrent theme in the literature is that of choice of intervention. Aminzadeh and Edwards examined preferences for different types of mobility aids between Italian and British Canadians [25]. Participants spoke about the shift in the status of canes in modern society from prestigious and fashionable devices in earlier times to now being a symbol of ageing and frailty:

In the eighteenth century, canes were in fashion. But today, let’s be honest, no one wants to use a cane, unless he really needs it. It would be nice to go back to those days. Back home in my village [in Italy] everyone would carry a cane.—Italian Canadian [25].

Acknowledging that the use of aids is appropriate and acceptable is often determined by the expectations of others from the same culture. For example, Italian and British Canadians differed in their opinions in their use of canes. Amongst the British, the prevalent view was that older people made their own decisions about whether or not to use a cane. On the other hand, Italian seniors described physicians as the most influential referent vis-a-vis health decisions [25]. Although the use of mobility aids may allow a greater range of choices, they were often judged by others as a symbol of loss of independence [36]. In a similar fashion, Italian Canadians accepted bathroom aids as promoters of safety and independent living, and even those who rigorously rejected walking aids expressed the need for such devices. Yet, British Canadians spoke about bathrooms aids as being things imposed on them, often by close members of their family:

My daughter brought a bath mat home unbeknown to me and put it in the bathtub and said, ‘Now, mother use it’.—British Canadian [25].

This may indicate a difference between private and public acknowledgement of the need to use assistive devices within different cultures.

Similarly, when exercise is offered as a fall-prevention intervention, personal choice about participation in exercise classes appears influenced by social and cultural norms and expectations. To improve uptake of exercise interventions, one paper suggested individuals be offered choices both in terms of type of exercise on offer and whether the intervention is delivered as an individual or as a group-based activity [30]. On the one hand, group training provides a safe environment for enjoyable and sociable activities:

It is so nice to be here because something is wrong with all of us, my husband said. And that is how life is for all of us. Because we see someone equal to ourselves..—Participant in exercise intervention [32].

Yet, it is also suggested that many older people actually dislike group-based training [26] as they are embarrassed by their lack of abilities. Classes need to be pitched at an appropriate level to allow for full participation, which is dependent on the level of physical ability:

Personally, I feel that it’s no good for me. No, first of all because it hurts! And to do what I’m supposed to do wears me out. It’s no fun always to be the loser. I like to be able to do what others do.—Refuser of exercise class [32].

Along with the environment, the style and composition of exercise classes also influenced the success or failure of a fall-prevention intervention. One study based in New Zealand [30] suggested that male members of an exercise intervention may have been embarrassed about participating in a Tai Chi class as they viewed the style and type of exercises as predominately female. In contrast, another paper suggested that older Chinese people in England valued Tai Chi classes [35]. Older Chinese people stressed the cultural importance of the intervention due to its Chinese origins. In contrast, any dance-based, more Westernised, intervention would be totally unsuitable for their culture:

What would my daughter think of me dancing? At my age, it’s a laughing matter. My dead husband would have been shocked.—Chinese respondent [35].

The high rate of Chinese participation in Tai Chi classes in the United Kingdom was also facilitated by increased social interaction after exercise. Many participants stayed behind and ate Chinese food offered once the class had finished:

I make friends and I enjoy the food after the class. It’s also a social thing; we catch up with news from others. It’s just like a big family.—Chinese respondent [35].

There also appears to be strong cultural influence on whether or not an older person views fall-prevention programmes in a positive or a negative manner. These influences are often linked with ideas of a metaphysical nature, such as fatalism. For example, in South Asian communities in the United Kingdom, there appears to be a cultural belief that the consequences of ageing are outside the control of an individual. Therefore, accepting that a fall is the will of God or Allah may become a significant barrier to behaviour change as falling is perceived to be out of the control of an individual:

Anything can happen at any time. I can’t say I have any fears. Things can happen. God knows..—South Asian respondent [29].

Chinese communities were reported to hold fatalistic beliefs towards falls and fall prevention. Falls are regarded as unavoidable and outside the control of an individual. However, in a crucial difference to South Asian communities, older Chinese people perceive it to be luck, rather than divine intervention, as to whether they fell or not [35]:

Maybe we Chinese always talk about luck [laughs]. Luck is important. You’re lucky if you win the lottery. You’re unlucky if you fall [laughs]. But seriously, it’s not that easy to say if falling has to do with bad luck. It could be bad luck, maybe, but I don’t know. Some people are more superstitious than others.—Older Chinese person [35].

Similar beliefs about fatalism were also evident in the Caucasian culture but were more linked to an acceptability of the ageing process rather than some metaphysical belief:

It’s your bones; they still get old [in relation to falls]. I don’t feel it’s any kind of exercise, it’s just your body ageing; you cannot do what you did when you were younger.—Caucasian female, age 66 [29].

### Psychosocial

This section examines two themes within our overarching concept of psychosocial considerations. These are (1) transforming identities and (2) defining the expert.

### Transforming identities

A major theme throughout the studies was that of how falling or being labelled at risk of falling serves to transform a person’s identity. A fall can have a devastating effect on independence, confidence, and quality of life [8]. Falling is associated with physical injuries, psychological trauma, functional impairments, and even death. One respondent described her fall as ‘the beginning of the end’ [25]. There is a reluctance among older people to be viewed as old and disabled [26]. Concerns with public appearance mean that often older people would walk without walking aids ‘just for show’. Aids designed to facilitate activities were viewed as indicators of lesser abilities and changed self [36]. Any restriction of activity led to feelings of frustration and a sense of loss, as older people could not continue to do what they may have done previously and needed to begin to rely more on others [31]. Although older people were aware of the possibility of falling and could identify situations that might increase their risk of falling, many frequently took calculated risks to stay in their home:

…because I’ve said…to myself…that you have to make sure you get out of bed every day…and that I’m able to take care of myself.—Respondent [32].

The need to maintain independence overrode any recommendations not to do so. The biggest fear of older people was being forced to give up their home, even if healthcare professionals had recommended some change in the home environment to reduce the risk of falling. Often, there were significant mismatches between the views of the healthcare professional and the older person with regard to ability to cope at home:

We watch it [functional activity] and our hearts are in our mouths… they’re functioning within their environment as best they can…they think they are doing a fine job.—Occupational therapist [21].

A main theme was the impact that falling had on day-to-day physical activity. Although walking aids and ‘sensible’ footwear may allow a greater range of choice, many aids were perceived as unwelcome, despite the assistance they afforded, and were negatively viewed as a marker towards loss of independence:

…I just watched them using them. They walked bent up. I mean I’m bent up by myself using that [referring to her wheeled walking aid], but not as bad as – maybe I look the same as somebody else, not as far as I could see, with people using a zimmer.—Respondent [36].

Barriers to direct intervention were predominately patient specific. Patient pride, willingness, and comprehension were all obstacles [14]. Loss of independence and confidence commonly result from falling, but a greater loss in the lives of older people comes from the loss of their identity as greater reliance on others becomes necessary [36]. Contact with social workers and healthcare professionals is not always perceived as empowering older people to live their daily lives and, in some cases, added to loss of meaningful identity. Many people did not appear to accept that their lives had been totally transformed by falling, but rather sought to make sense of themselves in changed circumstances [36]:

I want to be well. This is the thing that really annoys me. I mean I’m not enjoying being a semi-invalid. This is ridiculous, really ridiculous.—Housebound female [36].

### Defining the expert

Thirteen studies discussed expertise [13,21-23,25-28,31-34,37]. There are different hierarchies of expertise within different populations. The definition of ‘expert’ ranges from healthcare professionals [13,23,25,26,37] to families [22,28]. The studies suggest the existence of a further hierarchy within the category of expertise, where medical expertise (organisational) [25] appears to be favoured by healthcare professionals over the expertise of managing illness (individual) [22].

At an individual level, older people appear not to be considered by healthcare professionals as experts on fall prevention. They are assumed to lack the competence in identifying their own fall risk: ‘We don’t see the chronic ill elderly people as experts in their own area…’ (administrator in falls clinic) [37]. Conversely, in a study examining the views of older people about fall prevention, one man suggested that older people do have the expertise to manage themselves [26]. Following advice to stop climbing a ladder, he stated, ‘I have climbed this ladder for 50 years, don’t tell me that I will fall’.

The way that healthcare professionals offer fall advice and prevention programmes was often viewed as insulting and dictatorial by older people who still saw themselves as experienced and competent in their day-to-day functioning: ‘You should be very careful about the way you would approach old men and tell them that they might need to participate in this…’ (interviewee denying the need to undertake a fall-prevention intervention) [26]. Despite this clear insistence, healthcare professionals appear not to accept that fallers or older people can be experts. They are considered to lack the competency to identify their own propensity to fall and also take individual responsibility for their treatment. However, some older people did not think that healthcare professionals were actually interested in their well-being. Falls were perceived to be a minor ailment, something trivial: ‘No. You know…my GP [general practitioner], he don’t take notice’ (male not attending intervention) [34].

There appears to be little agreement between healthcare professionals regarding whether or not family members are able to properly identify fall risk. In one paper, family members were considered by healthcare professionals to potentially have such expertise [37], whereas in a paper examining the attitudes of older people towards advice given by family members, patients themselves may dispute such expertise, with one older lady stating, ‘…if my daughter advises me to use a cane, it goes in one ear and out the other’ [25]. Studies also address the expertise of staff working in healthcare organisations [13,22,23,26,37]. One paper suggested that some staff lack suitable fall-prevention training and also feel unclear about the depth of knowledge held by colleagues in other parts of the healthcare system [13]. Physicians felt that that exposure to geriatric training during medical school had a lasting influence on their approach to fall risk evaluation and management:

Some time in my first year of residency we got a crash course in geriatrics. And the thing that really stuck with me was if they break their hip they have a huge mortality.—Physician [22].

However, one study discussed a holistic approach to fall prevention and suggested that working with individuals who have fallen requires different expertise, such as observation, communication, and the ability to engage with the older person [23]. Yet, the use of a holistic fall assessment screening tool, which altered the nurse–patient relationship to that of prevention partnership, did not reposition the nurse–doctor hierarchy and suggested a continuing presence of different hierarchies of expertise within the healthcare profession, as the doctor always made final decisions about care despite not having undertaken a risk assessment themselves:

Some of the doctors are still very set and this is what they are going to do…And I’m afraid I can’t break that…I try flipping things under their nose saying, ‘Hey did you know about this?’ And ‘Do you know about that?’—Nurse [23].

## Discussion

The aim of this synthesis was to identify and explore factors that influence the successful implementation of fall-prevention programmes. Our synthesis identified three overarching key concepts. These were practical considerations, adapting for community, and psychosocial.

Our findings suggest that factors underpinning the successful implementation of fall-prevention programmes are complex. No single factor can be identified as a key facilitator. Most of the studies reported multiple barriers to fall prevention. This suggests that certain barriers and misconceptions may need to be addressed prior to participation in a fall-prevention programme [38]. Clearly, the type and delivery of an exercise programme needs to be tailored to fit individual preferences, as some individuals prefer to exercise alone or as part of a group or at home as opposed to a community setting. Support from family, friends, peers, and healthcare professionals is considered critical to promoting and maintaining engagement with any fall-prevention interventions. Patient choice in accepting an intervention is framed by the physiological and psychological impacts of the intervention and also by the social and cultural structures in which the patient is living [39]. Thus, social and cultural categories of ageing appear to shape both expectations of growing older and the treatment or prevention of falls [40].

Patient concordance with any fall-prevention intervention is crucial to a successful outcome in preventing falls. The adoption of holistic and patient-centred practices with older people being active participants rather than passive recipients is vital to achieve implementation. However, a hierarchy of expertise appears to remain in healthcare, with the physician still considered the overall ‘expert’ in any healthcare ‘relationship’. This imbalance can result in a failure by healthcare professionals to take into consideration the views and experiences of older individuals, leading to recommendations not being taken up [38]. A key challenge would appear to be how healthcare professionals can make older people aware of their potential risk of falling without causing distress or denial of a problem [18].

A major barrier to the successful implementation of fall-prevention programmes is the differing perceptions of fall risk amongst the older population, families, and healthcare professionals [18]. Tensions persist between the balance of power, expertise, and independence in the relationships between the older person, their families, and healthcare professionals. Some older people may express attitudes that increasing fall risk is symbolic of ageing, diminishing competence and independence, and that these are simply an inevitable part of ageing. In this rationale, no intervention can prevent falls from happening. Such a fatalistic view of falls is a significant barrier to behaviour change. If an older person believes they have little control over what happens to them, they may see little point in engaging with preventative behaviours. Therefore, designing fall-prevention programmes that can be successfully implemented is challenging. On the one hand, some respondents clearly rejected interventions that stereotyped them as being frail, yet others valued interventions that involved contact with people of a similar age and ability [18].

Overall, the findings point to a wide range of factors that will influence the implementation of fall-prevention strategies, although the extent to which these can be taken into account in developing or delivering fall-prevention programmes is not clear. As with all research, the strengths and limitations of this synthesis must be highlighted. One of the strengths of this approach is the ability to identify concepts and themes from the individual studies by thematic analysis and develop further interpretations across the studies as a whole. Utilising a multidisciplinary approach involving clinical, social science, and methodological expertise promoted rigour in the study methods and findings [41].

Efforts to improve the identification of factors influencing the implementation of fall-prevention programmes require a greater understanding of the particular barriers and facilitators that help or hinder the closure of the research/practice gap. Qualitative research can contribute to this understanding and help interpret the findings of quantitative studies [39]. There are no universally agreed upon quality indicators for use with qualitative research. Therefore, the level of methodological detail required for publication remains unclear [42]. The lack of guidance regarding methodological reporting requirements means that it may be impossible to conclude whether limitations are due to poor study methods or poor reporting [43]. There is no one way of synthesising data, and authors of the papers included in this systematic review have offered a multitude of ways, from descriptive to conceptual. Therefore, in some ways this systematic review has been restricted by the number of papers in the synthesis that are mostly descriptive in content, which has made it more difficult to produce a highly conceptual synthesis.

Synthesis of qualitative research may be viewed as bringing together a sum of parts on a chosen theme. By doing so, the results in conceptual terms should be greater than drawing conclusions from individual studies alone [44]. It may be useful for such qualitative enquiries to be carried out and reported alongside measures of the performance of the interventions. Ideally, this would also include a way that relates the findings of attitudes to the performance programmes. As Finfgeld [45] suggests, the ultimate evaluative criterion, however, will be the ability of the findings of metasynthesis to improve clinical practice and inform healthcare policies. For example, this review may help the healthcare professional to be more informed about factors that serve as barriers and facilitators to the successful implementation of fall-prevention programmes. Communication between the older person and the healthcare professional may also be improved by a greater understanding of what programmes are more acceptable to an older person and thus be translated into more effective clinical practice with improved outcomes [46].

## Conclusions

There are a range of factors affecting the implementation of fall-prevention practices. These are related to older people, families, healthcare professionals, and healthcare systems. In order to improve the implementation of fall prevention, beliefs and behaviours at individual, organisational, and societal levels need to be addressed. It appears important in clinical practice to consult with older people in order to ascertain what changes they are prepared to make in order to reduce their fall risk [47]. If this consultation does not happen, then fall-prevention programmes will be less effective as maximum participation rates may not be achieved. As McInnes *et al.* (2003) suggest, more work on synthesising disparate studies on patient views and preferences are needed to inform evidence-based recommendations [38].

## Competing interests

The authors declare they have no competing interests.

## Authors’ contributions

SC contributed to the study design, data extraction, evidence synthesis, and editing. VG contributed to the study design, devised the search strategy, and undertook screening, data extraction, evidence synthesis, and editing. RG provided methodological advice and contributed to the evidence synthesis and editing. TJH devised the research strategy and undertook screening and editing. KB devised the search strategy and ran the searches. KS overviewed the search strategy criteria, acted as screening arbiter, and contributed to editing. All authors read and approved the final version of the manuscript.

## Supplementary Material

Additional file 1 Search Strategies for papers. Click here for file

Additional file 2 Quality appraisal of included studies (based on Wallace et al; 2004). Click here for file
