# Supplementary material for: Factors influencing the implementation of fall-prevention programmes: a systematic review and synthesis of qualitative studies
Source: Implement Sci. 2012 Sep 14;7:91. doi: 10.1186/1748-5908-7-91 (PMC3576261; doi:10.1186/1748-5908-7-91)
Supplement: Additional file 1 — Search Strategies for papers. [file 1748-5908-7-91-S1.doc]

**Additional File 1: Search Strategies for papers**

1 Accidental Falls/

2 (fall or falls or faller$1 or fallen).ti,ab.

3 1 or 2

4 exp Aged/

5 (senior$1 or elder* or older or old or oldest).ti,ab.

6 4 or 5

7 3 and 6

8 (prevent* or reduc* or manage*).ti,ab.

9 7 and 8

10 Program Evaluation/

11 Information Dissemination/

12 Barrier*.ti,ab.

13 evaluat*.ti,ab.

14 translat*.ti,ab.

15 feasibility.ti,ab.

16 integrat*.ti,ab.

17 implement*.ti,ab.

18 disseminat*.ti,ab.

19 adopt*.ti,ab.

20 10 or 11 or 12 or 13 or 14 or 15 or 16 or 17 or 18 or 19

21 9 and 20

22 limit 21 to yr="1980 -Current"

The search was devised for Ovid MEDLINE(R) In-Process & Other Non-Indexed Citations and Ovid MEDLINE(R) 1946 to Present. It was run on the 31-1-12. The strategy was adapted for other databases accordingly.
