# Supplementary material for: Factors influencing the implementation of fall-prevention programmes: a systematic review and synthesis of qualitative studies
Source: Implement Sci. 2012 Sep 14;7:91. doi: 10.1186/1748-5908-7-91 (PMC3576261; doi:10.1186/1748-5908-7-91)
Supplement: Additional file 2 — Quality appraisal of included studies (based on Wallace et al; 2004). [file 1748-5908-7-91-S2.doc]

**Additional File 2:** Quality appraisal of included studies (based on Wallace et al; 2004)

|  | **Is the research question clear?** | **Perspective of author clear?** | **Perspective influenced the study design?** | **Is study design appropriate?** | **Is the context adequately described?** | **Sample adequate to explore range of subject/settings?** | **Sample drawn from appropriate population?** | **Data collection adequately described?** | **Data collection rigorously conducted?** | **Data analysis rigorously conducted?** | **Findings substantiated / limitations considered?** | **Claims to generalisability follow from data?** | **Ethical issues addressed?** |
| --- | --- | --- | --- | --- | --- | --- | --- | --- | --- | --- | --- | --- | --- |
| **Aminzadeh &Edwards. 2008. [25]** | **Yes** | **Unclear** | **Unclear** | **Yes** | **Yes** | **Yes** | **Yes** | **Yes** | **Yes** | **Yes** | **Yes** | **Yes** | **Yes** |
| **Baker et al 2005. [13]** | **Yes** | **Yes** | **Unclear** | **Yes** | **Yes** | **Yes** | **Yes** | **Unclear** | **Unclear** | **Yes** | **Unclear** | **Yes** | **Yes** |
| **Bell & Stirling 2006. [23]** | **Yes** | **Yes** | **Yes** | **Unclear** | **Yes** | **Unclear** | **Unclear** | **Yes** | **Yes** | **Unclear** | **Unclear** | **Yes** | **Yes** |
| **Chou *et al* 2005. [22]** | **Yes** | **Unclear** | **Unclear** | **Yes** | **Yes** | **Yes** | **Yes** | **Yes** | **Yes** | **Yes** | **Yes** | **Yes** | **Yes** |
| **De Groot & Fagerstrom**  **2010 [32]** | **Yes** | **Yes** | **No** | **Yes** | **Yes** | **Yes** | **Yes** | **Yes** | **Yes** | **Yes** | **Yes** | **Yes** | **Yes** |
| **Dickinson et al**  **2011 [34]** | **Yes** | **No** | **Unclear** | **Yes** | **Yes** | **Yes** | **Yes** | **Yes** | **Yes** | **Yes** | **Yes** | **Yes** | **Yes** |
| **Evron *et al* 2009a. [37]** | **Yes** | **Yes** | **Yes** | **Yes** | **Yes** | **Yes** | **Yes** | **Yes** | **Yes** | **Yes** | **Yes** | **Unclear** | **Yes** |
| **Evron et al 2009b. [28]** | **Yes** | **Yes** | **No** | **Yes** | **Yes** | **Unclear** | **Yes** | **Yes** | **Yes** | **Yes** | **Yes** | **Yes** | **Yes** |
| **Fortinsky *et al* 2004. [14]** | **Yes** | **Unclear** | **Unclear** | **Yes** | **Yes** | **Yes** | **Yes** | **Yes** | **Yes** | **Yes** | **Yes** | **Yes** | **Yes** |
| **Hanson & Salmoni**  **2011 [24]** | **Yes** | **Yes** | **No** | **Yes** | **Yes** | **Yes** | **Yes** | **Yes** | **Yes** | **Unclear** | **Yes** | **Yes** | **Yes** |
| **Hawley 2009. [31]** | **Yes** | **Unclear** | **Unclear** | **Yes** | **Yes** | **Yes** | **Yes** | **Yes** | **Yes** | **Yes** | **Unclear** | **Yes** | **No** |
| **Horne *et al* 2009. [29]** | **Yes** | **Yes** | **Unclear** | **Yes** | **Yes** | **Yes** | **Yes** | **Unclear** | **Unclear** | **Unclear** | **Yes** | **Unclear** | **Yes** |
| **Horton & Dickinson**  **2011 [35]** | **Yes** | **No** | **Unclear** | **Yes** | **Yes** | **Yes** | **Yes** | **Yes** | **Yes** | **Yes** | **Yes** | **Yes** | **Yes** |
| **Hutton et al 2009. [30]** | **Yes** | **Yes** | **No** | **Yes** | **Yes** | **Yes** | **Yes** | **Yes** | **Yes** | **Yes** | **Yes** | **Yes** | **Yes** |
| **Mackenzie 2009. [21]** | **Yes** | **Yes** | **No** | **Yes** | **Yes** | **Yes** | **Yes** | **Yes** | **Yes** | **Yes** | **Yes** | **Yes** | **Yes** |
| **Nahm et al**  **2009 [33]** | **Yes** | **Yes** | **No** | **Yes** | **Yes** | **Yes** | **Yes** | **Yes** | **Yes** | **Yes** | **Yes** | **Yes** | **Yes** |
| **Stewart & McVittie**  **2011 [36]** | **Yes** | **No** | **Unclear** | **Yes** | **Yes** | **Yes** | **Yes** | **Yes** | **Yes** | **Yes** | **Yes** | **Yes** | **Yes** |
| **Vernon & Ross 2008. [27]** | **Yes** | **Yes** | **Unclear** | **Yes** | **Yes** | **Yes** | **Yes** | **Yes** | **Yes** | **Yes** | **Yes** | **Unclear** | **No** |
| **Yardley *et al* 2006 [26]** | **Yes** | **Unclear** | **Unclear** | **Yes** | **Yes** | **Yes** | **Unclear** | **Yes** | **Yes** | **Yes** | **Yes** | **Yes** | **Yes** |
